# Profiling ATM regulated genes in *Drosophila* at physiological condition and after ionizing radiation

**DOI:** 10.1186/s41065-022-00254-9

**Published:** 2022-10-21

**Authors:** Jun Liu, Tianyu Jin, Lanxi Ran, Ze Zhao, Rui Zhu, Gangcai Xie, Xiaolin Bi

**Affiliations:** 1 School of Medicine, Nantong University, Nantong, 226001 China; 2 College of Basic Medical Medicine, Dalian Medical University, Dalian, 116044 China

**Keywords:** ATM, X-ray irradiation, miRNA, lncRNA

## Abstract

**Background:**

ATM (ataxia-telangiectasia mutated) protein kinase is highly conserved in metazoan, and plays a critical role at DNA damage response, oxidative stress, metabolic stress, immunity, RNA biogenesis etc. Systemic profiling of ATM regulated genes, including protein-coding genes, miRNAs, and long non-coding RNAs, will greatly improve our understanding of ATM functions and its regulation.

**Results:**

1) differentially expressed protein-coding genes, miRNAs, and long non-coding RNAs in *atm* mutated flies were identified at physiological condition and after X-ray irradiation. 2) functions of differentially expressed genes in *atm* mutated flies, regardless of protein-coding genes or non-coding RNAs, are closely related with metabolic process, immune response, DNA damage response or oxidative stress. 3) these phenomena are persistent after irradiation. 4) there is a cross-talk regulation towards miRNAs by ATM, E2f1, and p53 during development and after irradiation. 5) knock-out flies or knock-down flies of most irradiation-induced miRNAs were sensitive to ionizing radiation.

**Conclusions:**

We provide a valuable resource of protein-coding genes, miRNAs, and long non-coding RNAs, for understanding ATM functions and regulations. Our work provides the new evidence of inter-dependence among ATM-E2F1-p53 for the regulation of miRNAs.

**Supplementary Information:**

The online version contains supplementary material available at 10.1186/s41065-022-00254-9.

## Introduction

The ataxia-telangiectasia mutated (ATM) protein kinase is a member of the Phosphoinositide-3-Kinase (PI3K)-like Kinase (PIKK) family, and plays a master role to regulate DNA damage response (DDR), especially at double strand breaks (DSBs) repair, to maintain genome integrity [1, 2]. Besides of DNA damage, ATM activation has been shown in connection with other stimuli, such as oxidative stress [3], ATP depletion, mitotic spindle, or metabolic stress, specifically inhibition of glycolysis, which is independent of DNA damage or oxidative stress [1, 2].

Mutation of *atm* gene in humans can cause a rare genetic disorder ataxia telangiectasia (A-T), which is characterized by radiation sensitivity, neurodegeneration, immunodeficiency, cancer predisposition, premature aging, and metabolic dysfunctions [4]. While complete loss of ATM kinase activity or functions does not cause lethality in humans, most of A-T patients die at 20 s or 30 s of age [4], mice with kinase-dead *atm* mutations are embryonic lethal [5]. In *Drosophila*, *atm*/*tefu* mutated flies have aberrant small head, significant telomere-telomere fusions in neuroblasts, and are sensitive to irradiation [6–9]. While loss of function *atm* allele, *atm *^*stg*^, is pupal lethal [10], and *atm *^*8*^ is a heat sensitive loss of function allele [7].

ATM resides predominantly in the nucleus and is activated rapidly by DSBs in the Mre11-Rad50-Nbs1 (MRN) dependent manner. ATM and ATR (A-T and Rad3-related, another member of PIKK family) phosphorylate more than 700 proteins in response to DNA damage [11]. When ATM is activated by oxidative stress, it can phosphorylate a specific set of proteins that only partially overlap with the phosphorylated proteins in response to the canonical DSBs [12]. In response to replication stress induced by mitomycin C (MMC) and hydroxyurea (HU) treatment, ATM and ATR regulate SUMOylation modification [13]. Thousands of SUMOylation sites and hundreds of phosphorylation sites were identified after treatment, and proteins SUMOylated and phosphorylated are in the overlapping network.

ATM is required for both adaptive and innate immunity, and plays a supportive role in V(D)J-recombination, the core of adaptive immunity [4, 14]. The V(D)J-recombination generates programmed breaks, these breaks are held together in a synapse by the Recombination Activating Genes (RAG) protein. ATM is recruited to RAG-induced DSBs and activated, cooperates with MRN complex, γH2AX, and 53BP1, to stabilize DSBs during V(D)J-recombination, to ensure correct end processing and ligation [14].

Unscheduled transcription and alternative pre-mRNA splicing induced by DNA damage signify great threat to genome integrity. R-loop misregulation can lead to DNA damage, transcription elongation defects and genomic instability. R-loop activates ATM signaling, impedes spliceosome organization, and enhances UV-triggered alternative splicing [15, 16]. ATM and PIKK members influence DNA damage induced transcription through post-translational modification of RNA polymerase II (RNA pol II), transcription elongation factors, and multiple transcription factors, to regulate RNA-dependent DNA damage repair [17, 18]. ATM and ATR phosphorylate components of core spliceosome, promote displacement of spliceosome [1, 18]. Moreover, ATM modulates miRNAs biogenesis by regulating miRNAs transcription, nuclear exporting, processing, and maturation. On the other side, ATM is regulated by miRNAs at post-transcriptional level. ATM-miRNAs work coordinately to regulate DNA damage response and tissue homeostasis [19]. ATM kinase interacts and phosphorylates the KH-type splicing regulatory protein KSRP, a key component in both Drosha and Dicer miRNA-processing complex, enhances KSRP-pri-miRNAs interaction and miRNAs maturation [20].

ATM is required for the maintenance of glucose metabolism. Most A-T patients succumb to metabolic syndromes in early age, show the tendency towards insulin resistance, glucose tolerance, high blood pressure etc. Mutation of *atm* gene causes loss of redox sensing, increased ROS level, and altered redox balance, which leads to mitochondrial dysregulation and changes of glucose metabolism [1, 4]. A-T patients also develop early-onset cerebellar neurodegeneration, the neurological defect which might be due to loss of redox homeostasis and dysregulation in DNA damage response, however its molecular basis is still not clear [1, 4].

Due to the large number of ATM substrates with different functional features, ATM affects many cellular processes. A systematic genome-wide analysis of genes that may be regulated by ATM will provide a valuable reference for the study of ATM. In this study, we investigated differentially expressed (DE) protein-coding genes, miRNAs, and lncRNAs in *atm* mutated flies at physiological condition and after ionizing radiation. We found that biological functions of DE genes are diversified, regardless of protein-coding genes or non-coding RNAs, and a lot of DE genes are closely related with metabolic process, immune response, DNA damage response or oxidative stress. These phenomena are persistent even after irradiation treatment. We identified miRNAs regulated by ATM, E2f1 and p53 during development and after irradiation, and provided evidence of a cross-talk regulation among them. We proved that most of irradiation induced miRNAs in *atm* mutated flies are closely related with DNA damage response.

## Materials and methods

### Fly genetics

All flies were maintained at 25 °C on standard corn meal unless specified. Fly lines used in this study were: *w *^*1118*^ (*wild-type*, Bloomington Drosophila Stock Center 3605), *atm *^*stg*^ (*atm*) [6, 10], *miR-274 *^*KO*^ (Bloomington Drosophila Stock Center 58904); *miR-956 *^*KO*^ (Bloomington Drosophila Stock Center 58941) [21], *miR-963 *^*sp*^ (Bloomington Drosophila Stock Center 61449); *miR-1007 *^*sp*^ (Bloomington Drosophila Stock Center 61490) [22], *tubulin*-Gal4.

### Ionizing radiation

Actively crawling third instar larvae of *w *^*1118*^ and *atm *^*stg*^ flies were collected and irradiated with 40 Gy, at a dose rate of 340 cGy/min, with X-RAD 320 iX at 320 kV (Precision X-Ray, Inc., North Branford, CT). The irradiated L3 larvae were recovered for 1 h before further experiments.

### RNA-seq

Actively crawling third instar larvae of *w *^*1118*^ and *atm *^*stg*^ flies were collected. There are three replicates in each group with about 30 larvae in each replicate. RNA was purified from irradiated and non-irradiated flies. Total RNA was extracted using Trizol with DNase treatment at the Beijing Genomics Institute (BGI Co., Ltd., China). The integrity of extracted RNA was assessed by Agilent 2100 Bioanalyzer (Agilent Technologies, Palo Alto, CA). The mRNA sequencing libraries were constructed with Illumina TruSeq Stranded mRNA Sample Prep Kit (Illumina, Cat. No.RS-122–2101), and paired-end 50 bp sequencing was performed using Illumina HiSeq 2000. The small RNA libraries were constructed with Illumina’s TruSeq Small RNA Sample Prep kit (Illumina, Cat. No. RS-200–0012), and single-end 50 bp sequencing was performed using Illumina HiSeq 2000. All RNA-Seq experiments encompass three biological replicates.

### RNA-seq data analysis

For mRNAs and lncRNAs analysis, raw sequencing data quality was assessed with FastQC (v2.0.1) (https://www.bioinformatics.babraham.ac.uk/projects/fastqc/) and trim_galore (v0.6.6) [23], and aligned to *Drosophila* melanogaster reference genome (dmel_r6.41_FB2021_04) using STAR (v2.7.9a). Annotated genes were assigned using annotations from Flybase (dmel-all-r6.41.gtf) [24] and featureCounts (v2.0.1) [25]. For miRNAs analysis, raw sequencing data quality was assessed using FastQC (v2.0.1). The expression level of annotated miRNAs was quantified by miRDeep2 [26] using the quantifier.pl script with the following parameters: quantifier.pl -m mature.fa -p hairpin.fa -r reads.fa -t dme_index -y filename. Sequence files (mature.fa, hairpin.fa) of annotated miRNAs were retrieved from the miRBase Release 22.1 [27]. Differentially expressed genes (DEGs) were identified by comparison of genes expression in *atm *^*stg*^ flies with *w *^*1118*^ flies at same experimental condition using R package DESeq2 (version 1.25.12) [28], and a gene is considered differentially expressed when the absolute log2-fold change is ≥ 1 and the adjusted *p*-value is < 0.05.

### Reverse transcription-quantitative PCR

Total RNA was extracted using Trizol and reverse transcribed with Evo M-MLV RT Kit with gDNA Clean for qPCR (Accurate Biology, AG11705) using Oligo dT(18 T)primer 5’ d(TTTTTTTTTTTTTTTTTT) 3’. Gene transcripts were quantified by quantitative PCR (qPCR) with SYBR Green Premix Pro Taq HS (Accurate Biology, AG11701) on a Roche LightCycler 96 with comparative Cq method, *rp49* was used as a reference gene, and relative expression change was calculated from three independent experiments.

### Function enrichment analysis

The Gene Ontology (GO) annotation, KEGG pathway annotation and enrichment analysis were conducted for differentially expressed protein-coding genes and experimentally validated miRNAs targeted genes, based on Gene Ontology database (http://www.geneontology.org/), and KEGG (Kyoto Encyclopedia of Genes and Genomes) (http://www.genome.jp/kegg/) with PANTHER [29] and DAVID (The Database for Annotation, Visualization and Integrated Discovery, https://david.ncifcrf.gov/) [30]. For GO analysis, biological processes (BP) and molecular functions (MF) were applied, GO terms with adjusted *p*-value < 0.05 were considered significant. Significantly enriched biological processes were displayed with R software ggplot2 package and Cytoscape software [31].

### Viability assay

Actively crawling third instar larvae were irradiated with the dosage of 0, 10, 20, 30, and 40 Gy, respectively, at a dose rate of 1.3 Gy/min in a X-Ray Biological Irradiator (X-RAD 320iX, Precision X-ray Inc). At least 100 larvae were treated for each genotype at each dosage, and experiments were repeated three times. Survival percentage was calculated as the number of viable adults divided by the number of irradiated third instar larvae. Significant differences between the values under designated experimental conditions were subjected to two-tailed Student’s t-tests. For all tests, a *P* value less than 0.05 was considered statistically significant.

## Results

**Fig. 1:**
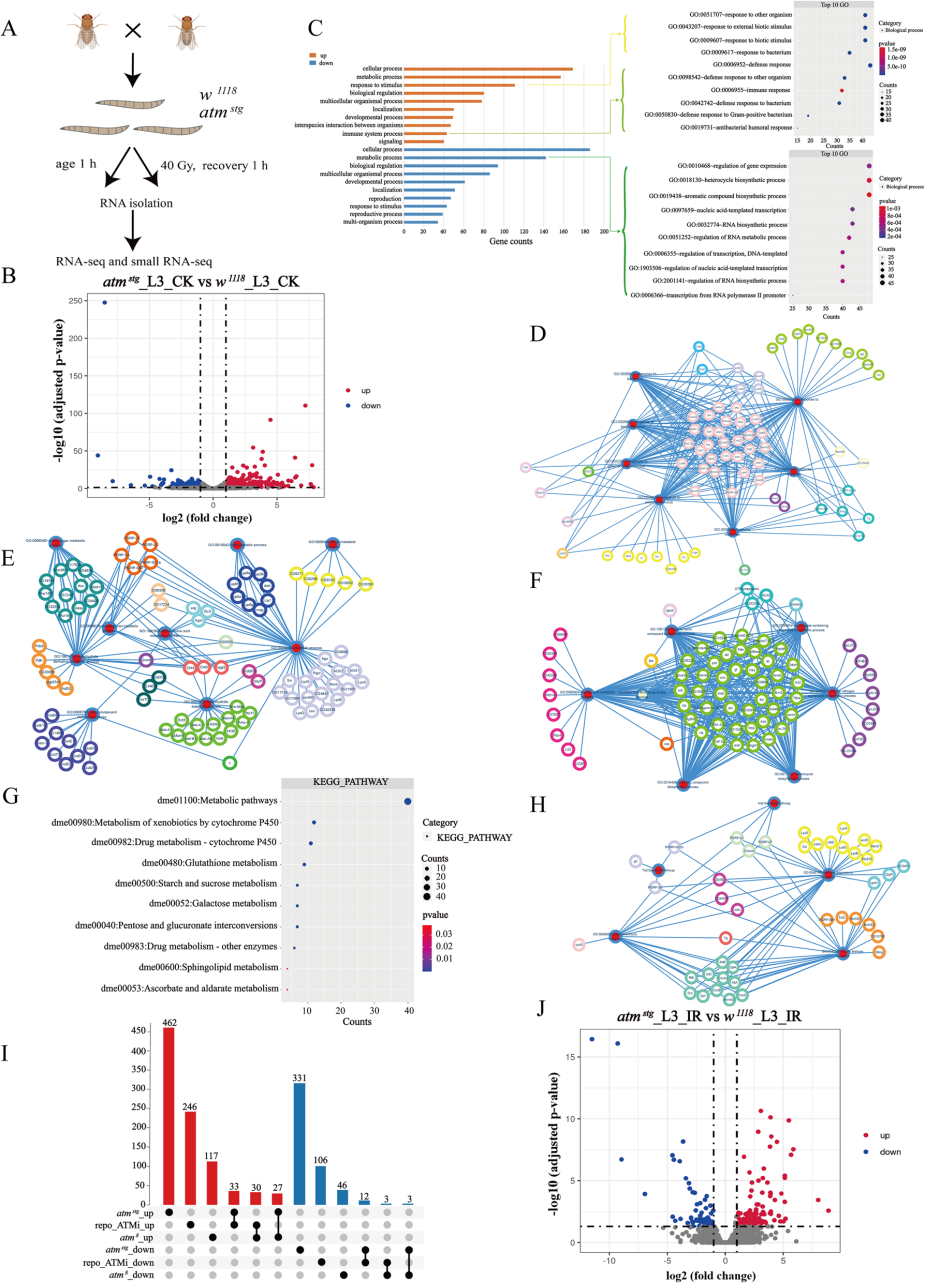
Differentially expressed (DE) protein-coding genes in *atm* mutated flies at physiological condition (CK) and after ionizing radiation (IR). **A** Experimental scheme. **B** Volcano plots of protein-coding genes differentially expressed between *atm* mutated flies and *wild-type* flies. up, up-regulated genes in *atm* mutant compared with *wild-type* flies; down, down-regulated genes in *atm* mutant compared with *wild-type* flies. **C** The top 10 GO terms of upregulated and downregulated protein-coding genes. **D** Functional gene clusters in response to stimulus. **E** Functional gene clusters in metabolic processes of upregulated genes. **F** Functional gene clusters in metabolic processes of downregulated genes. **G** KEGG pathways analysis. **H** Functional gene clusters in immune response. **I** UpSet plot of the number of overlapping genes between depicted genotypes of flies. **J** Volcano plots of protein-coding genes differentially expressed between *atm* mutated flies and *wild-type* flies after ionizing radiation

To explore potential protein-coding genes and non-coding RNAs regulated by ATM in *Drosophila*, we performed RNA-sequencing (RNA-seq) and small RNA-sequencing (miRNA-seq) on third instar larvae (L3) of *wild-type* (*wt*) and *atm* mutated flies (Fig. 1A). Raw sequencing data of RNA-seq was passed through stringent quality control, and aligned to the *Drosophila melanogaster* BDGP6 (dmel_r6.41_FB2021_04) using STAR (2.7.9a). In total, 1,025 million reads were collected at an average of 51.25 million reads per sample, range from 42.3 to 59.7 million reads, and mapped loci range from 68 ~ 96%. We identified 12,584 protein-coding genes with at least 10 reads using featureCounts (v2.0.1) (Table S1), which accounts for 95% of protein-coding genes in *Drosophila*. Differential expression of the protein-coding gene between the *atm* and *wild-type* flies was assessed using DESeq2 (Table S2), and a gene expressed with significant difference (fold change ≥  ± 2, adjusted *p* < 0.05) between the *atm* and *wild-type* flies in 3 replicates was regarded as differentially expressed. We identified 462 upregulated and 331 downregulated protein-coding genes in *atm* mutated flies when compared with *wild-type* flies (Fig. 1B, Table S3). The expression profile of differentially expressed genes was further verified by RT-qPCR for 10 randomly selected DE genes, including 5 upregulated genes and 5 downregulated genes (Figure S1). We also analyzed the tissue expression characteristics of these differentially expressed genes using tissue expression data from Flyatlas2 (Figure S2) [32].

To investigate the functional annotations of differentially expressed genes, we conducted Gene Ontology enrichment analysis with DAVID. In the GO analysis, the top 10 significantly enriched biological processes were identified in both upregulated and downregulated genes. The highly related biological processes that DE genes involved are cellular process, metabolic process, response to stimulus, developmental process, immune system process, reproduction etc. (Fig. 1C, Table S3). The cellular process and metabolic process are the top 2 biological processes in either upregulated or downregulated DE genes, there are more than 140 DE genes in each of them. And response to stimulus is the number 3 biological process in upregulated DE genes with 111 genes, and number 8 in downregulated DE genes with 43 genes, respectively (Fig. 1C, Table S3). Among DE genes in response to stimulus, they are highly enriched in biological processes such as immune response, stress, bacterial or biotic stimulus etc., and forms several highly connected functional gene clusters (Fig. 1D). Moreover, some DE genes are closely related with DNA damage response or oxidative stress. In upregulated genes, *agt* is required for DNA dealkylation [33], *tie* negatively regulate X-ray irradiation induced cell death [34], *TotC* is induced by oxidation, UV and bacterium treatment [35], *timeless* is involved into DNA replication checkpoint [36], and *corp* negatively regulate p53-dependent apoptosis [37]. While in downregulated genes, *eya* positively regulate DNA repair [36], *tfb4* is in response to UV and involved in nucleotide excision repair (NER) pathway [38]. Interestingly, some DE genes play roles in response to light stimulus, including *timeless* [36], *laza* [39] and *tfb4* [38] in upregulated genes, and *glass* [40], *inaC* [41], *ptth* [42], and *rh6* [36] in downregulated genes (Table S3).

As loss of human *atm* gene causes metabolic syndromes and neurodegeneration, we further investigated their functions in manually curated DE genes. In DE genes related with metabolic process, the top 3 biological processes in upregulated genes are protein metabolic process, cellular aromatic compound metabolic process, and carbohydrate derivative metabolic process. And in downregulated genes, they are cellular aromatic compound metabolic process, protein metabolic process, and nucleic acid metabolic process, respectively (Fig. 1E-F, Table S3). Many DE genes are involved in carbohydrate, lipid, amino acid, and RNA metabolic processes, suggesting the critical role that ATM protein kinase plays at metabolism. We also analyzed signaling pathways with KEGG of differentially expressed genes involved, and found that both upregulated genes and downregulated genes are highly enriched in metabolic pathways (Fig. 1G, Table S3). We further analyzed DE genes related with immunity, and found that some of them play important roles directly in humoral immune response, or innate immune response (Fig. 1H), in which *Charon*, *PGRP-LF*, *PGRP-SC1b*, and *PGRP-LC* are members in the Imd signaling pathway, and *gd*, *PGRP-SA*, and *PGRP-SC1b* are in the Toll signaling pathway, respectively (Table S3) [43–45]. Charon is an important mediator of PARP-1-dependent transcription in the innate immune pathway [46]. The *gd* gene is required for the Toll ligand produced by the proteolytic reaction [47]. A previous study performed microarray analysis using adult heads of a temperature sensitive *atm* allele (*atm *^*8*^), and identified 117 upregulated and 46 downregulated genes respectively, and found that decreased ATM kinase activity increases expression of innate immune response genes. Moreover, *atm* knockdown in glial cells resulted in neuronal and glial cell death, decreased mobility and lifespan. When a short hairpin RNA of *atm* gene was expressed by *repo*-Gal4 to knock-down *atm* gene in glial cells, genes expression in 3 ~ 5 days old adult flies were analyzed, there are 246 upregulated genes and 106 downregulated genes respectively, and innate immune response is the most significantly changed biological process [48]. We performed the comparative analysis. In upregulated genes, we identified 33 overlapping genes when compared with *repo*-*ATMi* allele, and 27 overlapping genes when compared with *atm *^*8*^ allele, respectively. While in downregulated genes, there are 12 overlapping genes when compared with *repo*-*ATMi* allele, and 3 overlapping genes when compared with *atm *^*8*^ allele (Fig. 1I), and most of overlapping genes are functional related with immune response.

To study ATM regulated genes in response to DNA damage, we treated the third instar larvae of both *wild-type* and *atm* flies with irradiation, and identified 129 upregulated genes and 61 downregulated genes (Fig. 1J, Table S4). We also analyzed the tissue expression characteristics of differentially expressed genes after irradiation (Figure S3). Among DE genes after irradiation, many of them are related with biological processes such as cellular process, metabolic process, developmental process, reproduction etc., although the number of differentially expressed genes was significantly reduced when compared with that of no irradiation treatment, which strengthen the previous suggestion that the fine tune of metabolic demands by communicating with surrounding milieu in response to stress is required in multicellular organisms [49].

### ATM regulates miRNAs biogenesis at both physiological condition and after ionizing radiation

As ATM plays an important role at regulating miRNAs biogenesis, we further investigated differentially expressed miRNAs in *atm* flies at physiological condition (NP) and in response to ionizing radiation (IR). Total RNA was prepared and small RNA-seq was performed. Raw sequencing data was processed with cutadapt to remove adapter sequence and generate the clean miRNA data. The miRNA reads were ranged from 38.4 to 54.6 million (Table S5), and mapped to genome with miRBase Release 22.1 using mirdeep2 (2.0.1.3). In total, we identified 281 annotated mature miRNAs with at least 10 reads (Table S5). The differential expression of miRNAs was assessed using DESeq2, and a miRNA gene expressed with significant difference (fold change ≥  ± 2, adjusted *p* < 0.05) between the *atm* and *wild-type* flies in 3 replicates was regarded as differentially expressed.

**Fig. 2:**
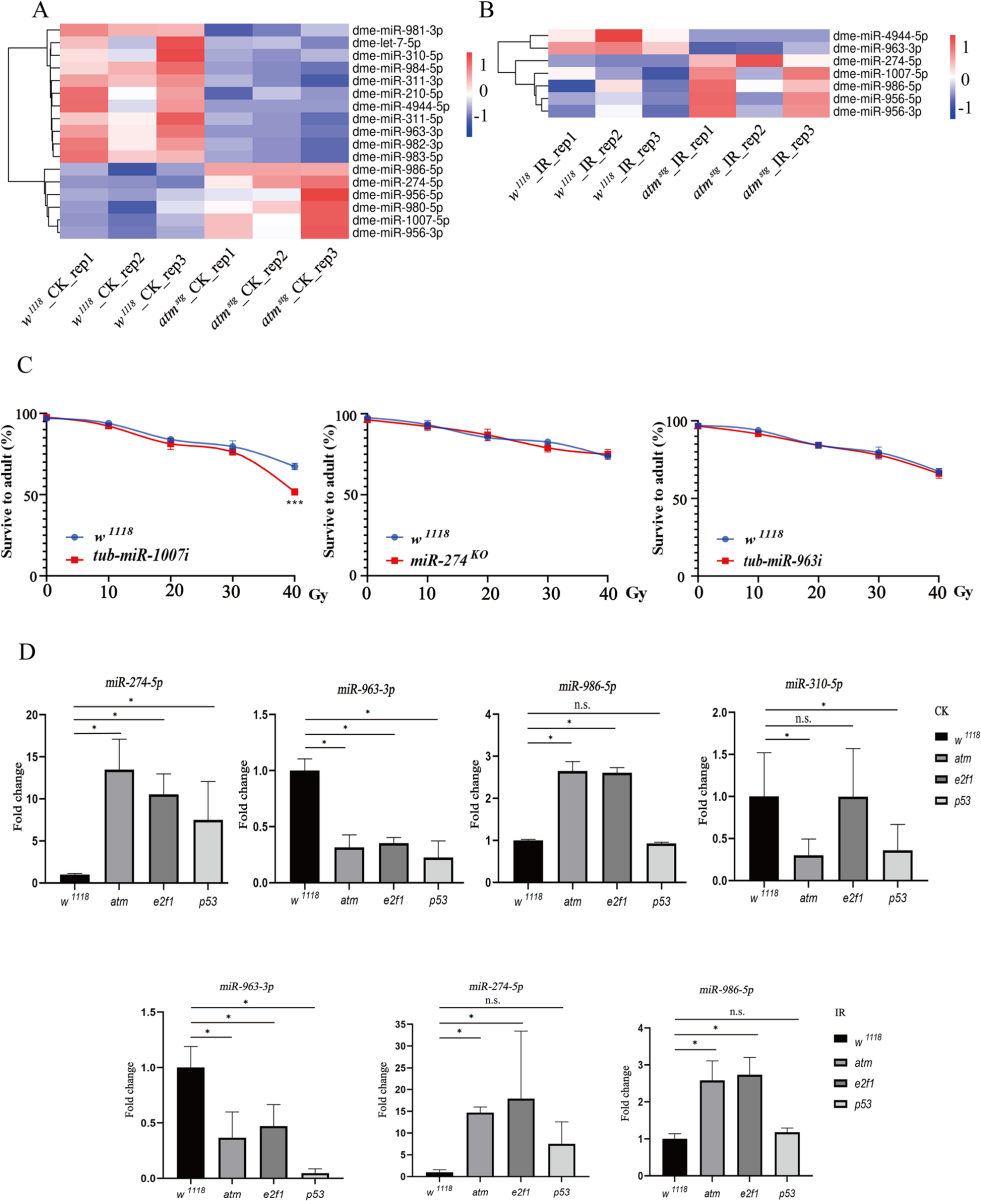
Differentially expressed miRNAs in *atm* mutated flies at physiological condition and after ionizing radiation. **A** Heatmap of miRNAs differentially expressed between *atm* mutated flies and *wild-type* flies. **B** Heatmap of miRNAs differentially expressed between *atm* mutated flies and *wild-type* flies after ionizing radiation. **C** Sensitivity of knock-out or knock-down flies of DE miRNAs to ionizing radiation. **D** Relative expression of DE miRNAs in *atm*, *p53* and *e2f1* mutated flies. * *p* ≤ 0.05; n.s., no significance. Error bars indicate SEM

Compared with *wild-type* flies, we identified 6 upregulated miRNAs, including *miR-274-5p*, *miR-956-5p*, *miR-956-3p*, *miR-980-5p*, *miR-986-5p*, and *miR-1007-5p*, and 11 downregulated miRNAs in *atm* flies, including *let-7-5p*, *miR-210-5p*, *miR-310-5p*, *miR-311-5p*, *miR-311-3p*, *miR-963-3p*, *miR-981-3p*, *miR-982-3p*, *miR-983-5p*, *miR-984-5p*, and *miR-4944-5p* (Fig. 2A, Table S6). In upregulated miRNAs with annotated functions, *miR-274-5p* coordinates nervous and vascular development in glia, regulates circadian behavior in astrocytes, and plays a role in response to hypoxia [50, 51]; *miR-956-5p* is related with muscular dystrophy and has the pro-virulence function [52, 53]; *miR-980-5p* regulates axon guidance and is related with muscular dystrophy, suppresses memory in adult brain, and is downregulated upon starvation and metabolic stress [52, 54, 55]. In downregulated miRNAs with annotated functions, *let-7-5p* forms a cluster with *miR-100* and *miR-125*, they play critical role during metamorphosis [56] and have diversified functions; *miR-210-5p* modulates circadian rhythms, locomotion, and lipid metabolism, and prevents neurodegeneration in retina [57–59]; *miR-310-5p*, *miR-311-5p* and *miR-311-3p* in the *miR-310*/*313* cluster transduce nutritional signals as a metabolic regulator, are required for normal synaptic transmission, and involved into Toll pathway mediated immune response by regulating expression of the antimicrobial peptide Drosomycin [60–62]; *miR-981-3p* negatively regulates anti-bacterial defense by reducing diptericin in IMD pathway [62]. While *miR-986-5p* and *miR-1007-5p* in upregulated genes, and *miR-963-3p*, *miR-982-3p*, *miR-983-5p*, *miR-984-5p*, and *miR-4944-5p* in downregulated genes have no annotated functions, their functions might be closely connected with ATM, which needs further investigation.

Furthermore, we identified 5 upregulated miRNAs, including *miR-274-5p*, *miR-956-5p*, *miR-956-3p*, *miR-986-5p*, and *miR-1007-5p*, and 2 downregulated miRNAs, including *miR-963-3p* and *miR-4944-5p*, in *atm* flies after irradiation (Fig. 2B, Table S7). We treated miRNA knock-out or knock-down flies with X-ray irradiation, looked at their survival rates to investigate whether they are sensitive to ionizing radiation. Our previous result has shown that *miR-956 *^*KO*^ and *miR-986 *^*KO*^ flies are highly sensitive to irradiation [63], while *miR-274 *^*KO*^ flies are not sensitive to irradiation, indicating that although *miR-274-5p* expression was increased after irradiation, it may not play a role at DNA damage response. We used *tubulin-Gal4* to drive ubiquitous expression of *miR-963 *^*sp*^ and *miR-1007 *^*sp*^, and found that *tublin-miR-1007i* (*tub-miR-1007i*) flies with *miR-1007-5p* knock-down are sensitive to irradiation, while *tublin-miR-963i* (*tub-miR-963i*) flies with *miR-963-3p* knock-down are not sensitive to irradiation (Fig. 2C). As miRNAs play a role to fine tune genes expression and activities, residual expression level of *miR-963-3p* may influence the DNA damage sensitivity, a knock-out mutant of *miR-963-3p* is required to test whether it plays a role at DNA damage response. We did not test *miR-4944-5p* as no allele is available.

We performed comparison analysis with DE miRNAs in *p53* or *e2f1* mutated flies [63]. At physiological condition, *miR-274-5p* was upregulated in *atm*, *p53* and *e2f1* mutated flies, *miR-963-3p* was downregulated in *atm*, *p53* and *e2f1* mutated flies, *miR-986-5p* was upregulated in *atm* and *e2f1* mutated flies, and *miR-310-5p* was downregulated in *atm* and *p53* mutated flies. After irradiation, *miR-963-3p* was downregulated in *atm*, *p53*, and *e2f1* mutated flies, *miR-274-5p* and *miR-986-5p* were upregulated in *atm* and *e2f1* mutated flies (Fig. 2D), suggesting a cross-talk regulation to miRNAs among ATM, E2f1 and p53 during development and after irradiation.

### Differentially expressed lncRNAs in *atm* mutated flies

**Fig. 3:**
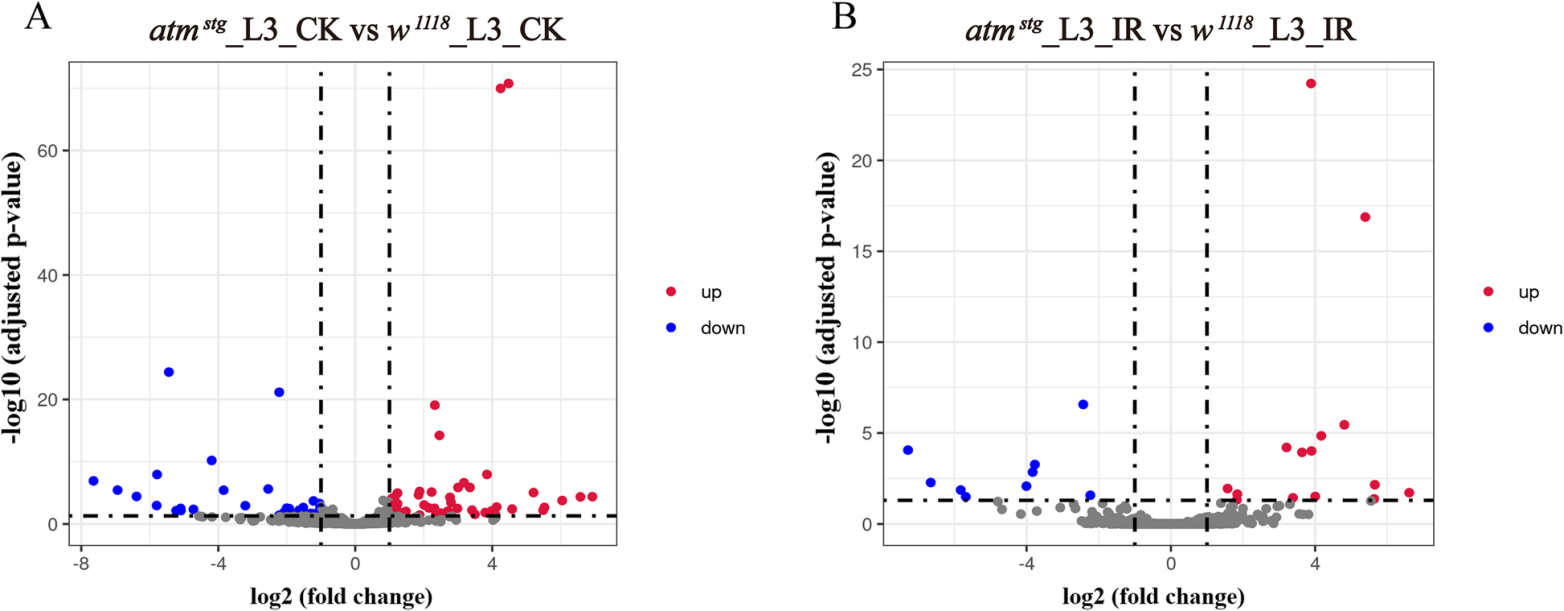
Differentially expressed lncRNAs in *atm* mutated flies at physiological condition and after ionizing radiation. **A** Volcano plots of lncRNAs differentially expressed between *atm* mutated flies and *wild-type* flies. **B** Volcano plots of lncRNAs differentially expressed between *atm* mutated flies and *wild-type* flies after ionizing radiation

We further investigated differentially expressed lncRNAs in *atm* flies at physiological condition and in response to ionizing radiation. RNA preparation, data processing, and identification of differentially expressed lncRNAs were performed as protein-coding genes. We identified 1128 lncRNAs with at least 10 reads (Table S8). Compared with *wild-type* flies, we identified 45 upregulated lncRNAs and 32 downregulated lncRNAs in *atm* flies at physiological condition (Fig. 3A, Table S9), and 15 upregulated lncRNAs and 9 downregulated lncRNAs in *atm* flies after irradiation (Fig. 3B, Table S10). Until now, very few lncRNAs in *Drosophila* have been studied, differentially expressed lncRNAs in *atm* mutated flies might play diversified roles as ATM kinase does. Three differentially expressed lncRNAs, including *CR31781*, which is upregulated in *atm* flies at physiological condition, and *CR43282* and *CR43356*, which are downregulated in *atm* flies at physiological condition, have been studied in systemic analysis. *CR31781* is involved in muscle lateral inhibition [64], and *CR43282* and *CR43356* knockout flies have reduced male fertility [65]. These three functional DE lncRNAs play different functions in *Drosophila*, such as in development and production, which is consistent with that of differentially expressed protein-coding genes and microRNAs.

## Discussion

The ATM protein kinase plays an important role and has diversified functions during development and in response to stress. Study of ATM regulated genes had been focused on post-translational modifications such as phosphorylation, somoylation etc. In this study, we systemically profiled differentially expressed genes in *Drosophila*, including protein-coding genes, miRNAs and lncRNAs, in *atm* mutated flies at physiological condition and after irradiation. We identified hundreds of differentially expressed protein-coding genes, and multiple miRNAs and lncRNAs. For those protein-coding genes and miRNAs with annotated functions, their functions are diversified and complex, span metabolism, immune response, multicellular organismal process, response to stimulus etc., which is consistent with our current understanding of the complex functions of ATM kinase.

The DNA damage response is highly conserved from invertebrates to mammals. Activation of systemic response is executed in response to DNA damage, including metabolic change, immune response etc., which plays very important role for the physiological adjustments for remodeling or regeneration of tissues and organs. Due to its simpler yet conserved genetics and physiology, *Drosophila* is of particular importance to investigate local and systemic interactions in response to stimulus. Moreover, compared with previous studies that showing proteins phosphorylated by ATM, our study provides a new angle of view to understand ATM functions and its downstream targets. Furthermore, by doing comparation analysis with our previous work studying miRNAs regulated by p53 and E2F1 [63], we identified miRNAs differentially expressed in *atm* mutated flies, *p53* mutated flies or *e2f1* mutated flies, and provide new evidence of inter-dependent regulation of miRNAs among ATM-E2F1-p53.

## Conclusions

Differentially expressed genes, including protein-coding genes and non-coding RNAs, in *atm* mutated flies have diversified functions and are highly related with metabolism, immune response, multicellular organismal process, response to stimulus etc. These phenomena are persistent after irradiation. There is a cross-talk regulation towards miRNAs by ATM, E2f1 and p53 during development and after irradiation.

## Supplementary Information

**Additional file 1:** **Figure S1.** RT-qPCR validation of DE protein-coding genes identified by RNA-seq. Validation of 5 upregulated and 5 downregulated protein-coding genes in *atm *mutated flies. Error bars indicate SEM.

**Additional file 2:** **Figure S2.** Tissue expression of differentially expressed protein-coding genes in *atm* mutated flies at physiological condition. **A** Heatmap of differentially expressed genes expressed in larval tissues. **B** Heatmap of differentially expressed genes expressed in adult female tissues. **C** Heatmap of differentially expressed genes expressed in adult male tissues.

**Additional file 3:** **Figure S3.** Tissue expression of differentially expressed protein-coding genes in *atm* mutated flies after ionizing radiation. **A** Heatmap of differentially expressed genes expressed in larval tissues. **B** Heatmap of differentially expressed genes expressed in adult female tissues. **C** Heatmap of differentially expressed genes expressed in adult male tissues.

**Additional file 4:** **Table S1.** mRNA counts.

**Additional file 5:** **Table S2.** Protein-coding genes sequenced.

**Additional file 6:** **Table S3.** Differentially expressed protein-coding genes in *atm* mutated flies.

**Additional file 7:** **Table S4.** Differentially expressed protein-coding genes in *atm* mutated flies after X-ray irradiation.

**Additional file 8:** **Table S5.** miRNA counts.

**Additional file 9:** **Table S6.** Differentially expressed miRNAs in *atm* mutated flies.

**Additional file 10:** **Table S7.** Differentially expressed miRNAs in *atm* mutated flies after X-ray irradiation.

**Additional file 11:** **Table S8.** lncRNA counts.

**Additional file 12:** **Table S9.** Differentially expressed lncRNAs in *atm* mutated flies.

**Additional file 13:** **Table S10.** Differentially expressed lncRNAs in *atm* mutated flies after X-ray irradiation.

**Additional file 14:** **Table S11.** Primers.

## Data Availability

The raw sequencing data generated in this study has been submitted to the NCBI BioProject database (https://www.ncbi.nlm.nih.gov/bioproject/) under accession number PRJNA809845. The custom code used is available at https://github.com/Liu970101/atm.

## Abbreviations

ATM: Ataxia-telangiectasia mutated
DDR: DNA damage response
DE: Differentially expressed
DEGs: Differentially expressed genes
DSB: Double strand breaks
GO: Gene Ontology
IR: Ionizing radiation
KEGG: Kyoto Encyclopedia of Genes and Genomes
L3: Third instar larvae
NP: Physiological condition
PIKK: Phosphoinositide-3-Kinase (PI3K)-like Kinase
RNA-seq: RNA-sequencing
