# Supplementary figures and images for: Profiling ATM regulated genes in Drosophila at physiological condition and after ionizing radiation
Source: Hereditas. 2022 Oct 21;159:41. doi: 10.1186/s41065-022-00254-9 (PMC9587650; doi:10.1186/s41065-022-00254-9)

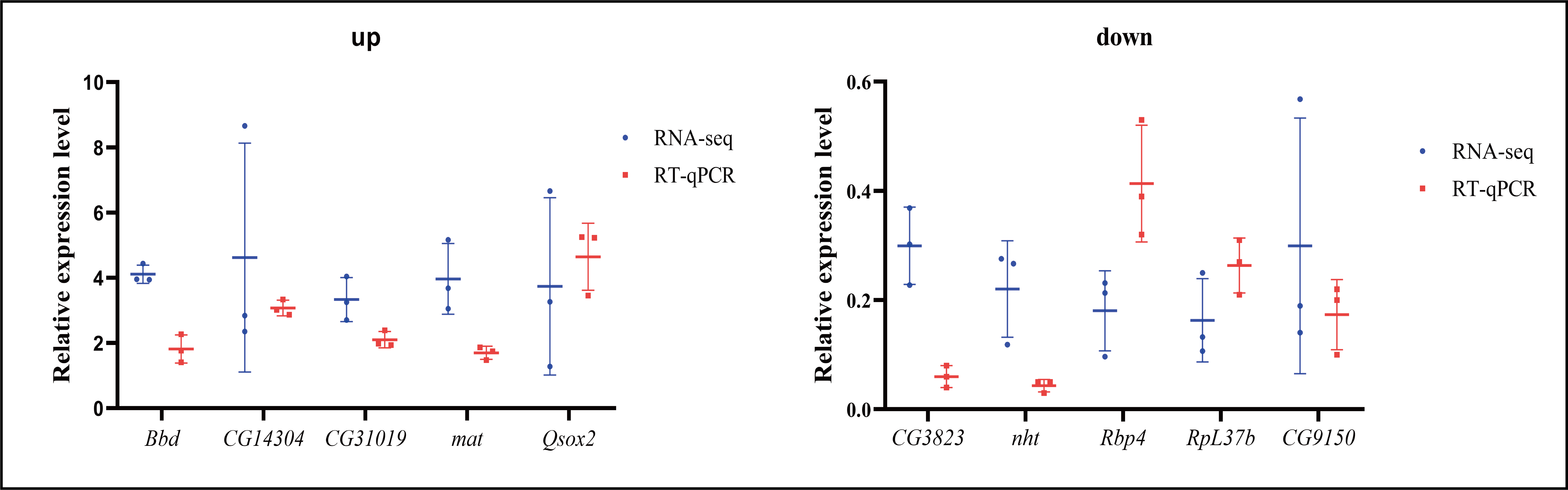

Supplement: Supplementary file 1 — Additional file 1: Figure S1. RT-qPCR validation of DE protein-coding genes identified by RNA-seq. Validation of 5 upregulated and 5 downregulated protein-coding genes in atm mutated flies. Error bars indicate SEM. [file 41065_2022_254_MOESM1_ESM.png]
