# Supplementary material for: Profiling ATM regulated genes in Drosophila at physiological condition and after ionizing radiation
Source: Hereditas. 2022 Oct 21;159:41. doi: 10.1186/s41065-022-00254-9 (PMC9587650; doi:10.1186/s41065-022-00254-9)
Supplement: Supplementary file 14 — Additional file 14: Table S11. Primers. [file 41065_2022_254_MOESM14_ESM.docx]

Table S11. Primers

| Gene name | Primer name | Primer sequence (5’-3’) |
| --- | --- | --- |
| Qsox2 | Qsox2 detect F | TTGCGATTCTTTGGTCCCGA |
|  | Qsox2 detect R | CAATTCGGCCAGTGTTGAGC |
| CG31019 | CG31019 detect F | CAGAATTGCACGCGGTGAAG |
|  | CG31019 detect R | CGGTTTCAATCCCTCTGCTC |
| Bbd | Bbd detect F | TTAACGAAAAGGAAACCGTCAC |
|  | Bbd detect R | GGCCAGTTCGATGACTATATCA |
| CG14304 | CG14304 detect F | GCAATTACCGCGACCACATC |
|  | CG14304 detect R | GGCGGGGCCATATTGAAGTA |
| mat | mat detect F | AGATTCAATGTGGAACTGGCTA |
|  | mat detect R | CTTTACGCCTTTGAGCATGTAT |
| CG9150 | CG9150 detect F | ACCAACGTAATGGGCGTGAT |
|  | CG9150 detect R | AGTTGAGCACCTGATGTCCG |
| CG3823 | CG3823 detect F | GACATGGCTGGCTATACGCT |
|  | CG3823 detect R | ACATACGACGGGCAGTTGAG |
| nht | nht detect F | GCTCGTCTGGAATCGGTGAA |
|  | nht detect R | CTGGGGCTAGTTGTTCTCCC |
| Rbp4 | Rbp4 detect F | ACTCAGACGACCGTGGAAAC |
|  | Rbp4 detect R | CCACATAGGTGACGAAGCCA |
| RpL37b | RpL37b detect F | AAGACGCACACCATCTGTCG |
|  | RpL37b detect R | GTACCGCATCCTTCCCGTTC |
